# Supplementary material for: Ancient recycled lower crust in the mantle source of recent Italian magmatism
Source: Nat Commun. 2019 Jul 19;10:3237. doi: 10.1038/s41467-019-11072-5 (PMC6642164; doi:10.1038/s41467-019-11072-5)
Supplement: Supplementary file 1 — Supplementary Information [file 41467_2019_11072_MOESM1_ESM.pdf]

# Supplementary Information

## Ancient recycled lower crust in the mantle source of recent Italian magmatism

Koornneef et al.

# Supplementary Note 1

**Why limestone assimilation can be excluded**

The rationale behind the methodology used in our study is to specifically select primitive olivine that crystallised and entrapped melt inclusions at depth (sub crustal levels). This ensures that any effects from crustal assimilation are avoided/minimised e.g., see [1]. The analyzed inclusions and their host olivine were thoroughly characterised in terms of major and trace element compositions. This work is especially aimed at determining the nature of the mantle source and partial melting controls, as also outlined in Nikogosian and van Bergen , 2010.

Discussion of the possible role of crustal assimilation and mantle enrichment processes below Italy stems back to the 1970s[2, 3]. Consensus was reached after two decades of work in the late 1990s e.g., [4]: (i) At some locations (e.g. Alban Hills, Vesuvius, San Venanzo) whole rock lavas show clear evidence of assimilation within the crust. This assimilation has obvious effects on the primary chemical and isotopic characteristics of the lavas e.g., [5], (ii) in contrast, Sr-Nd-Pb isotope compositions of high-MgO lavas erupted in the majority of volcanic centres in mainland Italy are not significantly affected by assimilation and are inherited from their mantle source[6, 7]. The petrography and geochemistry of the host lavas and the constituent minerals from Roccamonfina – Ernici have been extensively described in the literature[8, 9] and do not indicate a significant role of crustal assimilation at these locations.

Textural and compositional characteristics of the host lavas, constituent minerals and melt inclusions in our study are in agreement with this interpretation and the following observations provide further evidence against crustal assimilation:

1) Absence of textural evidence for assimilation

Assimilation of limestone will result in reactions with the magma that affect the petrology and petrographic textures of the volcanic products. This is recorded, for example, in high-potassium magmatism of the Alban Hills by the presence of calcite inclusions within phenocrysts, by sparry calcite in the groundmass, as well as late veinlets along grain boundaries and fractures[10, 11]. Furthermore, limestone assimilation will result in changing compositions of phenocrysts (olivine, cpx) crystallizing from the (contaminated) melt and/or unusual zoning (e.g. Ca-rich rims/overgrowths), or in corroded phenocrysts (e.g., K-feldspars with granular hypidiomorphic textures at Alban Hills). Finally, reactions with limestone would liberate excess $CO_2$[12] potentially to be included in crystallizing phenocrysts. The Roccamonfina-Ernici host rocks investigated in this study (Rocc-7 and Ern-4) do not record petrographic evidence of limestone assimilation; i) the rocks do not contain calcite in any form, nor any calc-silicate-rich reaction products (such as described for Vesuvius); ii) the olivine phenocrysts with trapped primary MIs do not show any unusual textural features; iii) primary olivine does not contain $CO_2$ rich fluid inclusions.

2) Primary compositions of host olivine

Assimilation of limestone would cause chemical modifications of lavas and constituent minerals. Olivine forsterite content (Mg/(Mg+Fe) *100 = Fo mol%) is a measure of how primitive or evolved a magma was when the olivine crystallised. The higher the Fo content, the more primitive the host magma. This is because fractional crystallization of olivine and clinopyroxene during magma evolutions will result in lowering the MgO while FeO increases. Limestone assimilation will result in a decrease in the Fo in olivine rims while CaO would increase[13, 14]. We observed these compositional effects at San Venanzo (See Supplementary Fig.1) but our Roccamonfina-Ernici olivines lack any

such Fo zonation towards the rim (See Supplementary Fig. 2). They have Mg#'s 88 – 90 mol.% typical for primary olivine. Hence, we agree with many previous workers (Boari et al., 2009; Conticelli et al., 2009 and several refs therein) that there is no evidence for significant crustal assimilation in the primitive lavas at Roccamonfina-Ernici.

3) Primary compositions of trapped melts

If limestone assimilation had occurred, it should be evident in the composition of the MIs. In addition to enrichment in CaO and relative depletions in $SiO_2$ (and other major components), assimilation would lower the concentrations of virtually all incompatible trace elements (ITE). The Roccamonfina/Ernici MI show the opposite. The Ca-rich inclusions carrying the unradigenic Pb component is enriched in many ITE (Fig. 4 in manuscript). Compared to Italian Mesozoic limestone from the Apennines [15], the ITE concentrations of Roccamonfina/Ernici MI are 10-100 times larger. Also, the ITE distribution pattern is very different from that of melts from the San Venanzo centre that experienced carbonate assimilation (Supplementary Fig. 3).

4) No evidence for mixing with Italian limestone in Sr-Nd isotope space

Italian limestone compositions reported in the literature define a relatively narrow range between $^{87}Sr/^{86}Sr$ = 0.707 and 0.708 [16, 17]. Assimilation with typical Italian limestone would thus not explain the Sr-isotope compositions of the MI. In Fig R5 we plot the Sr-Nd isotope variability of the MI together with a binary mixing model between inclusion ERN-363 (least affected by the exotic component, also used in Fig. 4) and a typical Italian limestone with $^{87}Sr/^{86}Sr$ = 0.707453; $^{143}Nd/^{144}Nd$ = 0.511824; Sr = 645 ppm; Nd = 9 ppm [9]. This simple model underscores the mismatch between the MI compositions and contamination with Italian crustal limestone (Supplementary Fig 4).

In conclusion, based on the textural and compositional characteristics of the host lavas, olivines and melt inclusions we can exclude the possibility that our MI compositions were influenced by limestone assimilation in the crust.

Supplementary Figures

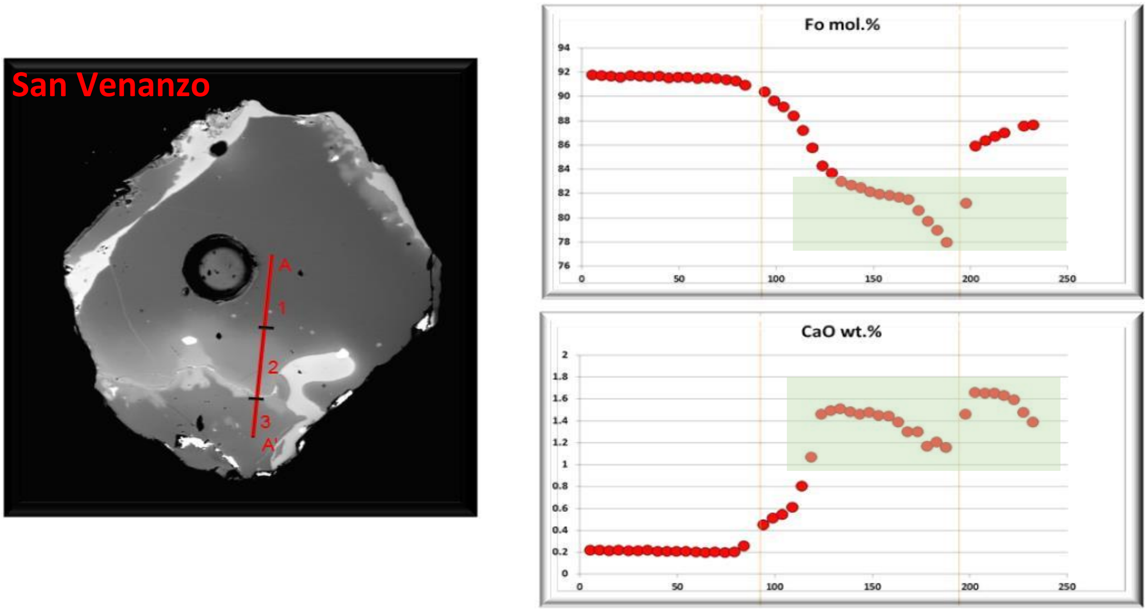


**Supplementary Fig 1.** Compositional profiles of olivine phenocrysts affected by assimilation at San Venanzo[18]

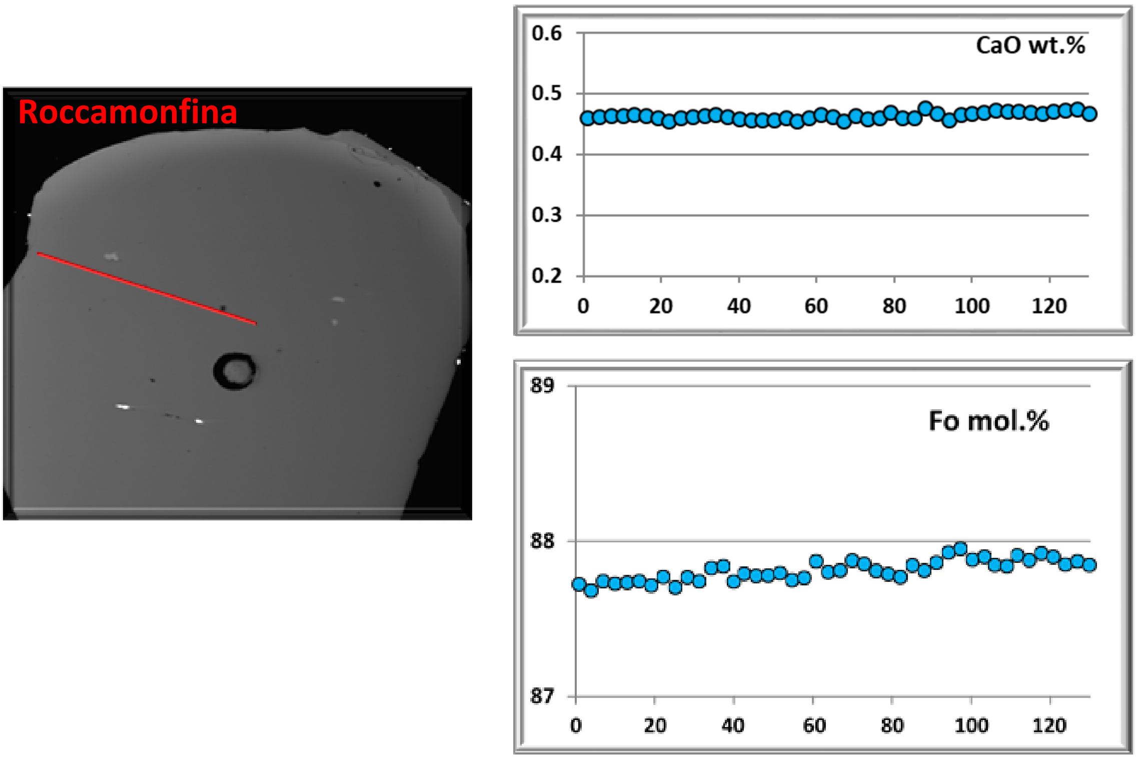


**Supplementary Fig 2.** Fo-rich olivines from Roccamonfina-Ernici do not show abnormal compositional zonation[19]

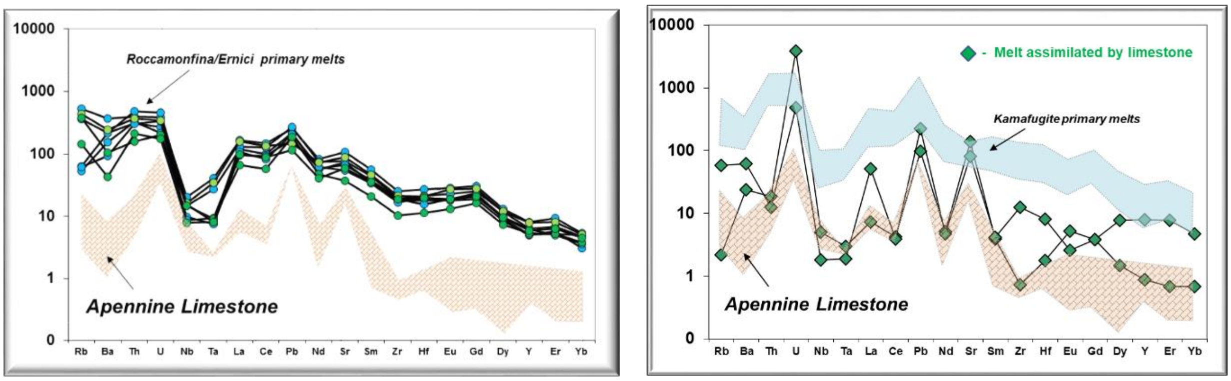


**Supplementary Fig 3.** Comparison of primary HKS melt inclusions from Roccamonfna/Ernici with Apennine Limestone. Trace element patterns of Roccamonfina-Ernici melt inclusions do not comply with limestone assimilation. Note that melt inclusions that assimilated limestone (San Venanzo, panel on the right) show much lower trace element contents. Data from[20].

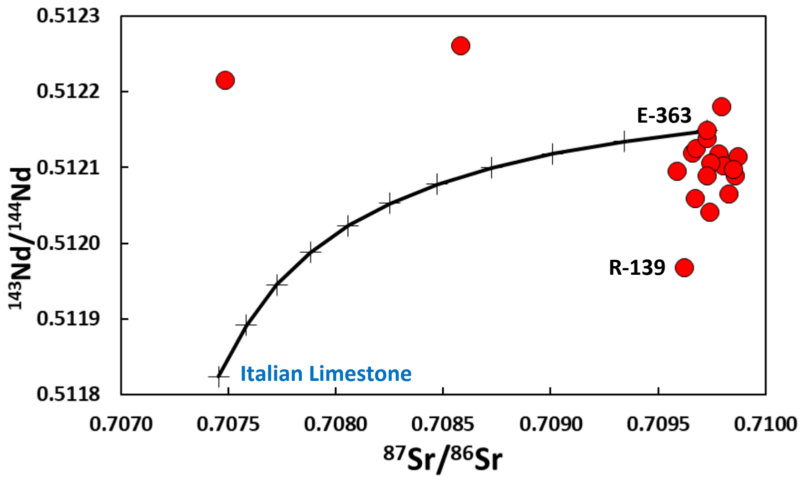


**Supplementary Fig 4.** Binary mixing model between a typical Italian limestone and melt inclusion ERN-363. Tick marks represent 10% fractions. Data source Supplementary Data 1.

## Supplementary References


1. Saal AE, Hart SR, Shimizu N, Hauri EH, Layne GD. Pb isotopic variability in melt inclusions from oceanic island basalts, Polynesia. *Science* **282**, 1481-1484 (1998).

2. Rogers NW, Hawkesworth CJ, Parker RJ, Marsh JS. The geochemistry of potassic lavas from Vulsini, central Italy and implications for mantle enrichment processes beneath the Roman region. *Contrib Mineral Petrol* **90**, 244-257 (1985).

3. Hawkesworth C, Vollmer R. Crustal contamination versus enriched mantle - $^{143}Nd/^{144}Nd$ and $^{87}Sr/^{86}Sr$ evidence from the Italian volcanics. *Contrib Mineral Petrol* **69**, 151-165 (1979).

4. Conticelli S. The effect of crustal contamination on ultrapotassic magmas with lamproitic affinity: mineralogical, geochemical and isotope data from the Torre Alfina lavas and xenoliths, Central Italy. *Chemical Geology* **149**, 51-81 (1998).

5. Peccerillo A. *Cenozoic Volcanism in the Tyrrhenian Sea Region*. Springer International Publishing (2017).

6. Conticelli S, Avanzinelli R, Ammannati E, Casalini M. The role of carbon from recycled sediments in the origin of ultrapotassic igneous rocks in the Central Mediterranean. *Lithos* **232**, 174-196 (2015).

7. Peccerillo A, Lustrino M. Compositional variations of Plio-Quaternary magmatism in the circum-Tyrrhenian area: Deep versus shallow mantle processes. *Geological Society of America Special Papers* **388**, p. 421-434 (2005).

8. Boari E, Tommasini S, Laurenzi MA, Conticelli S. Transition from Ultrapotassic Kamafugitic to Sub-alkaline Magmas: Sr, Nd, and Pb Isotope, Trace Element and $^{40}Ar$-$^{39}Ar$ Age Data from the Middle Latin Valley Volcanic Field, Roman Magmatic Province, Central Italy. *Journal of Petrology* **50**, 1327-1357 (2009).

9. Conticelli S, *et al.* Trace elements and Sr–Nd–Pb isotopes of K-rich, shoshonitic, and calc-alkaline magmatism of the Western Mediterranean Region: Genesis of ultrapotassic to calc-alkaline magmatic associations in a post-collisional geodynamic setting. *Lithos* **107**, 68-92 (2009).

10. Gaeta M, *et al.* Time-dependent geochemistry of clinopyroxene from the Alban Hills (Central Italy): Clues to the source and evolution of ultrapotassic magmas. *Lithos* **86**, 330-346 (2006).

11. Peccerillo A, Federico M, Barbieri M, Brilli M, Wu T-W. Interaction between ultrapotassic magmas and carbonate rocks: Evidence from geochemical and isotopic (Sr, Nd, O) compositions of granular lithic clasts from the Alban Hills Volcano, Central Italy. *Geochimica Et Cosmochimica Acta* **74**, 2999-3022 (2010).

12. Iacono-Marziano G, Scaillet B, Gaillard F, Pichavant M, Chiodini G. Role of non-mantle CO2 in the dynamics of volcano degassing: The Mount Vesuvius example. *Geology* **37**, 319-322 (2009).

13. Lustrino M, *et al.* Ca-rich carbonates associated with ultrabasic-ultramafic melts: Carbonatite or limestone xenoliths? A case study from the late Miocene Morron de Villamayor volcano

(Calatrava Volcanic Field, central Spain). *Geochimica et Cosmochimica Acta* **185**, 477-497 (2016).

14. Gaeta M, Di Rocco T, Freda C. Carbonate Assimilation in Open Magmatic Systems: the Role of Melt-bearing Skarns and Cumulate-forming Processes. *Journal of Petrology* **50**, 361-385 (2009).

15. Conticelli S, Marchionni S, Rosa D, Giordano G, Boari E, Avanzinelli R. Shoshonite and sub-alkaline magmas from an ultrapotassic volcano: Sr-Nd-Pb isotope data on the Roccamonfina volcanic rocks, Roman Magmatic Province, Southern Italy. *Contrib Mineral Petrol* **157**, 41-63 (2009).

16. Frijia G, Parente M, Di Lucia M, Mutti M. Carbon and strontium isotope stratigraphy of the Upper Cretaceous (Cenomanian-Campanian) shallow-water carbonates of southern Italy: Chronostratigraphic calibration of larger foraminifera biostratigraphy. *Cretaceous Research* **53**, 110-139 (2015).

17. Melluso L, D'Antonio M, Conticelli S, Mirco NP, Saccani E. Petrology and mineralogy of wollastonite- and melilite-bearing paralavas from the Central Apennines, Italy. *American Mineralogist* **88**, 1287-1299 (2003).

18. Ersoy Ö, Nikogosian IK, van Bergen MJ, Mason PRD. Phosphorous incorporation in olivine crystallized from potassium-rich magmas. *Geochimica et Cosmochimica Acta* **253**, 63-83 (2019).

19. Nikogosian IK, van Bergen MJ. Heterogeneous mantle sources of potassium-rich magmas in central-southern Italy: Melt inclusion evidence from Roccamonfina and Ernici (Mid Latina Valley). *Journal of Volcanology and Geothermal Research* **197**, 279-302 (2010).

20. Nikogosian I, Van Bergen MJ, Chaneva S. Multiple origins of carbon in Italian kamafugite melt. *Mineralogical Magazine (Goldschmidt Abstracts 2013)* **77**, 1851 (2013).
