# Supplementary material for: Ancient recycled lower crust in the mantle source of recent Italian magmatism
Source: Nat Commun. 2019 Jul 19;10:3237. doi: 10.1038/s41467-019-11072-5 (PMC6642164; doi:10.1038/s41467-019-11072-5)
Supplement: Supplementary file 3 — Description of Additional Supplementary Files [file 41467_2019_11072_MOESM3_ESM.pdf]

Description of Additional Supplementary Files

File Name: Supplementary Data 1

Description: Geochemical data on host lavas, host olivine, and melt inclusions from Roccamonfina-Ernici.
